# Editorial: 360° VR in sport and physical activity – it is very real

**DOI:** 10.3389/fpsyg.2023.1342533

**Published:** 2023-12-13

**Authors:** Aden Kittel, Ian Cunningham, Paul Larkin

**Affiliations:** ^1^Institute for Health and Sport (IHES), Victoria University, Melbourne, VIC, Australia; ^2^School of Applied Sciences, Edinburgh Napier University, Edinburgh, United Kingdom; ^3^MSA Research Centre, Maribyrnong Sports Academy, Melbourne, VIC, Australia

**Keywords:** 360° video, virtual reality, sport, immersive video, athletes

## 1 Introduction

With the advancement of technology, 360°VR (also termed immersive video) has been integrated within several sporting and physical activity settings. 360°VR differs to Virtual Reality (VR) as it presents real-world footage captured from a 360° video camera, whereas VR generates simulated artificial scenarios similar to a video game (Kittel et al., 2020a). Within contemporary research, 360°VR has predominantly been used to explore potential methods to develop perceptual-cognitive skills such as decision-making (Panchuk et al., 2018), with the simulated environment provided by 360°VR found to increase representativeness compared to traditional 2D video presented on a computer or television, and also more enjoyable (Kittel et al., 2020b). 360°VR has also been investigated in motor skill development, such as teaching volleyball (Paraskevaidis and Fokides, 2020), learning procedures in climbing and water safety skills to children and adolescents (Gänsluckner et al., 2017; Araiza-Alba et al., 2021). Positive outcomes associated with these investigations further support the use of 360°VR to be used as a training and assessment tool mainly due to the stronger fidelity 360°VR provides (Kittel et al., 2019). With further advances in technology and continued use of 360°VR in the field, there is scope for wider applied advances of this technology as a pedagogical tool for motor learning and skill development.

Building on the established literature, the goal of this Research Topic is to further develop and promote the use of 360°VR in sporting and physical activity environments while discerning its efficacy and utility. We have welcomed articles evaluating different applications of 360°VR in sport and physical activity that address diverse opinion positions and research themes: (i) practice design principles for skill acquisition and adaptation, (ii) pedagogical tool for reflection, (iii) diagnostic tool for assessing decision-making, and (iv) assess and monitor visual gaze and motor behavior in sport performance. This Research Topic contains four original manuscripts, including three original research pieces (Boyer et al.; Höner et al.; Taupin et al.) and one opinion piece (Lindsay et al.).

## 2 Practice design principles for skill acquisition and adaptation

There is an increased need for sport trainers to design practice environments to give learners more ecological solutions for developing opportunities for action and performance adaptations. In their opinion article, Lindsay et al. provide insights into key practice design considerations of 360VR for skill development, through a constraints-led approach perspective. Considerations are focused on three key areas, including constrain to afford, representative design, and repetition without repetition. This paper provides insights into how this technology can be used as an Action Observation tool.

## 3 Pedagogical tool for reflectivity

Sport officials' training environments are inherently challenged by limited representative tools to develop officiating skills. Boyer et al. qualitatively explored how young referees use 360°VR videos and the relationship with their viewing experience. Through grounded analysis with 12 student football referees, results indicated different types of focus on the game and also their peer referee's activity. Importantly, this study furthered the understanding of 360°VR technology as a reflective tool through different types of immersion including empathetic, simulation and exploratory involvement.

## 4 Diagnostic tool for assessing decision-making

Objective and evidence-based diagnostic tools for assessing potential talent predictors in athletes is a key agenda within talent identification and development. Höner et al. evaluated the development and applicability of a 360°VR diagnostic tool to assess decision-making skills of youth soccer players. The study provided 48 youth players with evolving footage of a soccer passage of play, terminating in the central midfield area. At the conclusion of the video, participants were asked what the best option to continue the play was. Results found participants had a positive immersion experience, further supporting the fidelity and representativeness of 360°VR. In addition, the specific results highlighted the ability for the 360°VR tool to differentiate age and level of performance of participants. Further, higher performing individuals, were six time more likely to have a chance of playing in one of the top four professional German leagues. Overall, the findings indicated the ability to develop and validate the use of a 360°VR tool to assess decision-making performance of youth soccer players.

## 5 Assess and monitor visual gaze and motor behavior

Video-based tests are often used to study anticipation and decision-making in sport whereas 360°VR viewing modes have received limited attention. Taupin et al. conducted a comparison study exploring the impact of viewing perspective on the gaze behavior and head excursions in a 360°VR boxing anticipation task. Overall, 32 participants viewed footage of a single punch sequence, in either 360°VR or 2-dimensional perspective. The findings indicated there were shorter fixations and greater head excursions in the 360°VR condition. Overall, the results indicated the potential advantage for using 360°VR to recreate more naturalistic real-world examples, further supporting the use of 360°VR technology to assess and monitor motor skill performance.

## 6 Final considerations

The articles included in the Research Topic “*360*°*VR in sport and physical activity – it is very real*” provide new insights into how 360VR technology can be implemented in different sporting environments for skill development. It is anticipated that this will continue to lay the foundation for more empirical research, and applied use of this technology in different areas.

## Author contributions

AK: Conceptualization, Writing—original draft, Writing—review & editing. IC: Conceptualization, Writing—original draft, Writing—review & editing. PL: Conceptualization, Writing—original draft, Writing—review & editing.
